# Evolution of genomic variation in the burrowing owl in response to recent colonization of urban areas

**DOI:** 10.1098/rspb.2018.0206

**Published:** 2018-05-16

**Authors:** Jakob C. Mueller, Heiner Kuhl, Stefan Boerno, Jose L. Tella, Martina Carrete, Bart Kempenaers

**Affiliations:** 1 Department of Behavioural Ecology & Evolutionary Genetics, Max Planck Institute for Ornithology, Seewiesen, Germany; 2 Sequencing Core Facility, Max Planck Institute for Molecular Genetics, Berlin, Germany; 3 Department of Conservation Biology, Estación Biológica de Doñana – CSIC, Sevilla, Spain; 4 Department of Physical, Chemical and Natural Systems, University Pablo de Olavide, Sevilla, Spain

**Keywords:** Strigiformes, colonization, demography, population genomics

## Abstract

When a species successfully colonizes an urban habitat it can be expected that its population rapidly adapts to the new environment but also experiences demographic perturbations. It is, therefore, essential to gain an understanding of the population structure and the demographic history of the urban and neighbouring rural populations before studying adaptation at the genome level. Here, we investigate populations of the burrowing owl (*Athene cunicularia*), a species that colonized South American cities just a few decades ago. We assembled a high-quality genome of the burrowing owl and re-sequenced 137 owls from three urban–rural population pairs at 17-fold median sequencing coverage per individual. Our data indicate that each city was independently colonized by a limited number of founders and that restricted gene flow occurred between neighbouring urban and rural populations, but not between urban populations of different cities. Using long-range linkage disequilibrium statistics in an approximate Bayesian computation approach, we estimated consistently lower population sizes in the recent past for the urban populations in comparison to the rural ones. The current urban populations all show reduced standing variation in rare single nucleotide polymorphisms (SNPs), but with different subsets of rare SNPs in different cities. This lowers the potential for local adaptation based on rare variants and makes it harder to detect consistent signals of selection in the genome.

## 1. Introduction

Urbanization is one of the most prevailing causes of habitat transformation and biodiversity loss worldwide [1]. Urbanization typically involves a set of environmental changes including habitat fragmentation, increased temperature, air, noise and light pollution, altered resource availability and reduced predation or parasite pressure [2]. Not all species respond negatively to these transformations, and there are many examples of successful urban colonization. Among birds, urban colonization has been related to intraspecific variation in fear of humans: it has been proposed that urban life selects for individuals with a smaller flight initiation distance upon approach [3,4]. This and other phenotypic changes associated with urbanization [5], combined with the lower predation pressure in urban areas [6], can affect not only the demography but also the dispersal propensity of individuals, with consequences for the dynamics and spatial structure of rural and urban populations.

Colonization of urban habitats by animals and plants is ideal to study recent adaptive and non-adaptive processes at the genome level. The habitat of city centres and suburban areas and their historical development appear to be similar across a wide geographical range and thus represent repeated natural experiments to study evolution in action [2,7]. Being exposed to a set of environmental changes during urbanization should lead to strong selection in the colonizer, whose signatures should be identifiable locally in the genome [5]. However, urban colonization typically goes hand-in-hand with demographic perturbations such as population bottlenecks or fragmentation, which can lead to similar genome signatures as adaptive processes, although across all loci [2,8]. Studying repeated urban settlements by the same species might help to disentangle random genome-wide demographic effects from local deterministic effects based on selection. While similar selective pressures among different cities could lead to the same selected regions or regulatory pathways in the genome, standing genomic variation (in particular the presence or absence of rare variants) on which selection can act may vary randomly among those cities if they were colonized independently. It is thus important to describe the genomic population structure and to understand the demographic effects on the genome and its consequences on potential adaptive processes. Models of the demographic history of the populations can then be used as null models when scanning for genomic targets of selection.

Urban colonization by birds has occurred on an ecological time scale, dating back more than 150 years (in case of the European blackbird *Turdus merula*; [9]) to only a few decades (e.g. in the dark-eyed junco *Junco hyemalis;* [10] or the monk parakeet *Myiopsitta monachus*; [11]). Because of the short time scale and the short geographical distance between neighbouring urban and rural populations, population structure is expected to be weak. Furthermore, only those genomic characteristics that change sufficiently strongly during such short time scales are potentially informative for elucidating the recent demographic history. Founder effects during the colonization and subsequent inbreeding typically lead to (i) reduced genetic diversity, but also to (ii) increased linkage disequilibrium (LD) between genetic markers, whereby such LD should decay with distance between markers over multiple generations. Recent studies show that population-level genomic sequence data can reveal weak population structures [12] and can be used to extract LD values for any distance class [13].

Here, we analyse the population structure and demographic history of burrowing owls (*Athene cunicularia*) in recently colonized Argentinian cities in comparison to neighbouring rural populations. Burrowing owls, a species typically associated with open grasslands in North and South America, have been reported as breeding birds in South American suburban areas for a few decades [3]. Burrowing owls have been historically associated with fossorial mammals such as prairie dogs (*Cynomys* sp.) in North America or the plains viscachas (*Lagostomus maximus*) in South America, whose burrows are used for nesting [14]. However, the owls of the Southern range evolved the ability to excavate their own burrows for nesting [14], which may have facilitated moving into the urban environment [6]. We do not know when this behaviour first emerged, but today all urban burrowing owls and most rural individuals in our study area excavate their own nests (less than 25% of the rural pairs use burrows of plains viscacha for nesting; M. Carrete 2018, unpublished data). The colonization of urban areas by these owls appears to be quite successful given their relatively high population densities in comparison to rural populations [15]. Reduced predation pressure has been described as one of the driving forces of this success [6]. Moreover, changes in behavioural traits due to selection or selective immigration have been documented in urban populations. Risk perception—measured as flight initiation distance—is an individually consistent and heritable trait and differs consistently between urban and rural individuals [16,17]. Information from previous studies of urban and rural burrowing owls thus suggests that the species represents a suitable study system for rapid and successful adaptation to the new urban environment.

In this paper, we present a high-quality genome assembly of the burrowing owl and we re-sequenced 137 individuals from three urban–rural population pairs to address the following questions: (i) what is the population structure of urban and rural populations of burrowing owls? We expected a weak structure given the supposedly recent urban colonization and the high dispersal ability of birds; (ii) were the different cities independently colonized, or were they colonized from a single source population that spread across all cities? (iii) what are the contemporary gene flow patterns among neighbouring populations? (iv) does the demographic history differ between the young urban populations and the rural populations? We discuss the implications of our results in terms of our ability to detect local adaptation and signatures of selection in the burrowing owl genome.

## 2. Materials and methods

### (a) Sampling

**Figure 1.**
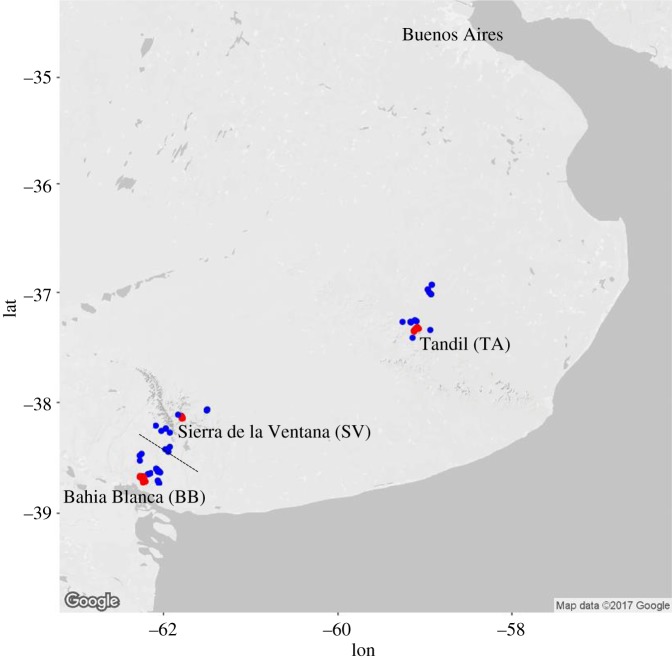
Sampling locations of urban sites (red) and rural sites (blue). The dotted line indicates the division between the two rural sampling sites BB_rural_ and SV_rural_ halfway between the cities.

As part of a long-term study, a population of burrowing owls has been individually marked, monitored and blood-sampled since 2006 in and around Bahia Blanca, Argentina [3,15]. Between 2012 and 2016, breeding burrowing owls have also been sampled in and near two additional Argentinian urban settlements of the pampas, Sierra de la Ventana and Tandil, located 77 and 314 km linear distance from Bahia Blanca, respectively. Breeding habitats of the owls were classified as either rural or urban following the criteria in Carrete & Tella [3] and Rebolo-Ifran *et al*. [4]. ‘Urban nests' were defined as those located in private and public gardens and along streets usually within 10–100 m of inhabited buildings. ‘Rural nests’ were those found outside the city border in grasslands and pastures of wide-ranging livestock where human presence and activities were minimal. For our study, we defined *a priori* seven sampling sites for the three urban–rural comparisons (figure 1): Bahia Blanca urban 1 and 2 (BB1_urban,_ BB2_urban_), Bahia Blanca rural (BB_rural_), Sierra de la Ventana urban (SV_urban_) and rural (SV_rural_), Tandil urban (TA_urban_) and rural (TA_rural_). From each of these sampling sites, we randomly selected 20 breeding individuals from 2012 to 2016, excluding first-degree relatives, except for SV_rural_ where we only sampled 17 individuals (total *n* = 137 blood samples). For the largest and more intensively monitored city (Bahia Blanca) we sampled 20 individuals from each of two spatially separated suburban areas (BB1_urban_ and BB2_urban_) to test the robustness of demographic inference based on simulations of 20 individuals (see below). Both urban sites were matched with BB_rural_. Below, we refer to each sample site as a ‘population'. For the reference genome, we used an additional blood sample from an adult male from the rural area around Bahia Blanca. All blood samples were stored in ethanol before analysis in the laboratory.

### (b) Genome assembly and annotation

We extracted DNA from the reference male blood sample using a standard phenol–chloroform protocol. The sample was used to construct a 500 bp insert Illumina Truseq2 paired-end sequencing library, a Nextera gel-free mate-pair library (insert size peak at 2500–3500 bp) and a 10 000 bp Nextera size-selected mate-pair library. DNA sequencing was performed using a 2 × 100 bp sequencing protocol on an Illumina Hiseq2500 system at the Sequencing Core Facility of the MPI for Molecular Genetics in Berlin.

After adaptor removal and quality clipping as well as removal of PCR-duplicated read pairs, we assembled the sequencing reads using the IDBA assembler [18] followed by the Newbler v. 3 assembler (Roche Diagnostics, Basel, Switzerland). This hybrid assembly strategy is described in more detail in the online electronic supplementary material, text S1. We obtained a total sequencing coverage of about 46-fold.

We annotated protein-coding sequences by alignment of all avian protein sequences of the NCBI protein database (date: 2015-09-22; 1 786 861 proteins) against the burrowing owl genome using SPALN v. 2.1.2 [19].

### (c) Sequencing, reference mapping and single nucleotide polymorphism calling of urban and rural individuals

We extracted DNA from all blood samples from the urban and rural populations with the Blood QuickPure kit (Macherey-Nagel) applying a pre-digestion with Proteinase K in Digsol buffer. All 137 birds were individually sequenced with the Illumina HiSeq 2500 technique using a 200- to 300 bp insert paired-end library with 125 bp read length at the Sequencing Core Facility of the MPI for Molecular Genetics in Berlin.

For each individual, we mapped reads against the reference genome using Bowtie2 [20]. We called variants using the GATK HaplotypeCaller in gvcf mode and assigned individual genotypes by a joint genotype calling of all samples [21]. The single nucleotide polymorphisms (SNPs) were then quality-filtered following fixed rules from the GATK best practices recommendations [21]. Additionally, to avoid SNP calls from mis-assembled paralogous genome regions, SNPs were only included if read depth was smaller than the mean coverage plus five standard deviations across all SNPs. For subsequent analyses, we excluded Z-chromosomal SNPs, SNPs with minor allele count of one (singletons) and SNPs with more than 12 missing genotypes across the 137 individuals.

### (d) Data analysis

For each individual, we calculated overall heterozygosity (% of all SNPs that are heterozygous) using vcftools [22]. The same tool was used to count the total number of SNPs and to calculate allele frequencies in each population. The expected number of shared and non-shared SNPs for urban–urban population pairs under random sampling was estimated by permuting the individuals between the populations 20 times and averaging the resulting SNP numbers. We use these metrics to characterize the colonization history, in particular to assess founder effects.

To assess population structure, we calculated *F*_ST_ values [23] between all population pairs and performed a principal components analysis (PCA) on the genotypes of all individuals using the R package SNPRelate [24,25]. We used a subset of 2 292 644 SNPs, such that they showed a composite genotypic LD of less than 0.5 within windows of 50 kb to avoid the influence of linked SNP clusters on these analyses. We tested the significance of *F*_ST_ values by randomly permuting individuals between the populations 1000 times and comparing the observed *F*_ST_ value with the simulated *F*_ST_ values. The between-population genetic variance (as a proportion of the total variance) and its significance were estimated in an analysis of molecular variance (AMOVA) and a distance-based redundancy analysis (dbRDA) framework using the R packages pegas and vegan, respectively [26,27]. For both methods, we used the proportion of alleles that differ between two individuals as a measure of their genetic distance. The dbRDA analysis tests population differentiation after accounting for the effect of geographical distance (longitude and latitude effects). In addition, we explored population structure by a discriminant analysis of principal components (DAPC) using the R package adegenet [28]. To avoid overfitting with too many PCs, we determined the optimal number of PCs based on the difference between the probability of reclassification into the pre-specified populations and the probability of reclassification into randomly permuted clusters (‘a-scores'; electronic supplementary material, figure S1). The optimal number (highest a-score) was 24 PCs, accounting for 26% of the total genetic variance. In the cluster analyses, individuals of a given population that are assigned to a neighbouring population indicate contemporary dispersal.

**Figure 2.**
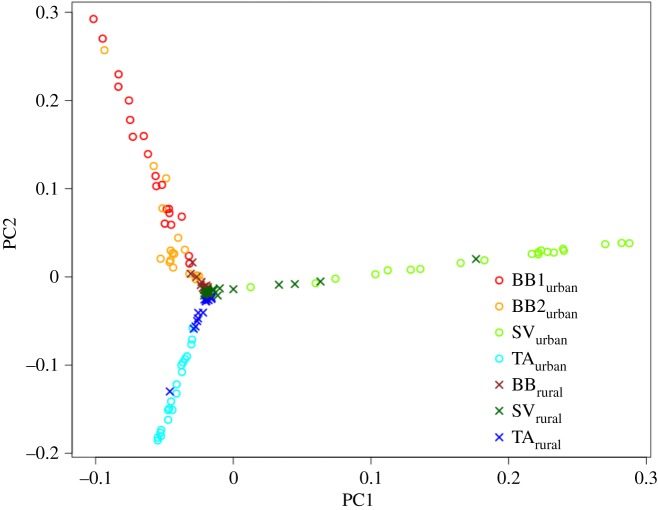
All individuals plotted on the first two principal components of a PCA on SNP genotypes (see Materials and methods). PC1 accounts for 1.6% and PC2 for 1.4% of the total variance.

Within each population, we tested for ‘isolation-by-distance' using Mantel tests between identity-by-state values (calculated using SNPRelate) and geodesic distances (calculated using the R package geosphere; [29]) implemented in the R package ecodist [30]. We further tested the combined longitude and latitude effect at different geographical scales in a dbRDA approach (see above). We also calculated the genetic distance of urban individuals to rural ones as the Euclidian distance from the position of the individual on the PCA plot (see above and figure 2) to the centre of all rural birds (defined as the median PC1 = −0.019 and the median PC2 = −0.017 of the rural individuals). We then plotted these genetic distances on the city map to assess—by visual inspection—whether the city area was sequentially colonized by founders.

We used an approximate Bayesian computation (ABC) method to estimate ancestral dynamics of effective population sizes (*N*_e_) within each population. First, we simulated 200 000 datasets of 20 individuals using the software package PopSizeABC [13] and the coalescent-based simulator scrm [31]. We simulated 20 Mb-long sequences assuming a mutation rate of 4 × 10^−9^ and a recombination rate of 2 × 10^−8^. We chose those values as representative for the unknown rates in the burrowing owl because they lie between the genome-wide average values for chicken and passerines [32–35]. We allowed effective population size to change randomly by a factor of 10 between 32 and 316 228 individuals at 21 time points between 3 and 500 generations before the present.

For each simulated dataset, we then extracted the allele frequency spectrum (AFS) and average genotypic LD values of 20 distance bins ranging from 50 kb to 17 Mb, whereby we excluded SNPs with minor allele frequency less than 0.15. We included long-range LD values to estimate recent population sizes because theory predicts that LD between markers of more than 10 Mb distance on chromosomes with a recombination rate of 2 cM Mb^−1^ is affected by the population size 2.5 generations ago [13,36]. Second, we extracted the observed AFS and LD values from SNP data of the 16 scaffolds that were longer than 20 Mb, representing 79% of the assembled genome. Third, we compared the simulated summary statistics with the observed ones to identify the most similar simulations using the R package abc [37]. These simulations were selected with the simple rejection method and an acceptance rate of 0.00005. We plotted *N*_e_ over time assuming an average generation time of 2.5 years. Following the calculations of Boitard *et al*. [13], the prediction error for 2000 random samples of the 200 000 simulations was around 0.33 for all time intervals. This indicates that the average difference between true and estimated population sizes is three times smaller than if estimated with the prior distribution of population sizes, resulting in a reasonable power to distinguish demographic histories (electronic supplementary material, figure S2).

## 3. Results

### (a) Genome assembly, individual sequencing and single nucleotide polymorphism calling

Our assembly of the burrowing owl (based on one adult male from the rural surrounding of Bahia Blanca) comprised 1144 Mb in 34 184 contigs with an N50 length of 64.1 kb. Scaffolding and splitting potential mis-assemblies resulted in scaffold N50 length of about 6.4 Mb (in total 1104 scaffolds, confirmed by consistently and uniquely mapped mate-pair coverage larger than 4-fold). After the final reference-assisted scaffolding step, the scaffolds were placed into 447 superscaffolds with an N50 length of 42.1 Mb. Ninety-nine per cent of the burrowing owl assembly (or 58 of the largest scaffolds) were assigned to *Gallus gallus* chromosomes or linkage groups. We annotated 17 858 protein-coding gene models in the genome assembly, which were on average 22.6 kb long and comprised 35% of the genome size (including introns). These gene models comprised 157 528 protein-coding exons covering 2.3% of the genome size. The burrowing owl genome browser can be found at http://public-genomes-ngs.molgen.mpg.de and is publicly available.

Sequencing coverage of the 137 burrowing owls ranged from 8.7-fold to 42.5-fold (median: 17.0-fold) and did not differ between urban and rural birds (*t*-test: *t*_131_ = −0.34, *p* = 0.73). After quality-control and singleton-filtering and after excluding Z-chromosomal SNPs, the total dataset contained 11 114 442 SNPs (on average 1 SNP per 100 bp).

### (b) Population structure

Overall, there were fewer SNPs in the urban populations than in the nearby rural populations (paired *t*-test: *t*_3_ = −4.96, *p* = 0.016; table 1). The number of SNPs in the urban populations of BB1, BB2, SV and TA respectively were 7%, 4%, 12% and 9% lower in comparison to the number of SNPs of their rural counterpart (table 1). Urban populations predominantly showed a lack of rare SNPs in comparison with their nearest rural population (electronic supplementary material, figure S3). Comparisons among the urban populations revealed that they differed in rare SNPs more than expected by random sampling (electronic supplementary material, figure S4). However, mean heterozygosity did not differ between urban and rural populations (paired *t*-test: *t*_3_ = 0.43, *p* = 0.70; table 1).

**Table 1.** Number of SNPs, heterozygosity, geographical distance between individuals and tests for isolation-by-distance.

	no. SNPs	heterozygosity (mean ± s.d.)	mean geographical distance (metre ± s.d.)	** **Pearson correlation coefficient (95% CI)**^a^**
BB1_urban_^b^	7 336 788	0.110 ± 0.013	1287 ± 815	−0.315 (−0.428 to −0.234)
BB2_urban_^b^	7 605 854	0.111 ± 0.009	1918 ± 1218	−0.271 (−0.343 to −0.193)
SV_urban_	6 561 678	0.114 ± 0.004	1090 ± 829	−0.119 (−0.245 to −0.012)
TA_urban_	7 083 000	0.114 ± 0.003	1991 ± 1529	−0.280 (−0.348 to −0.212)
BB_rural_	7 916 698	0.111 ± 0.004	12 469 ± 8408	−0.292 (−0.358 to −0.193)
SV_rural_	7 478 491	0.116 ± 0.002	28 435 ± 20 168	0.050 (−0.037 to 0.132)
TA_rural_	7 798 361	0.111 ± 0.009	24 244 ± 16 568	−0.312 (−0.357 to −0.271)

^a^Mantel test: correlation between identity-by-state values and geographical distances (see Materials and methods for details).

^b^Pearson correlation coefficient (95% confidence interval (CI)) of Mantel test for BB1_urban_ and BB2_urban_ combined: −0.308 (−0.349 to −0.276).

*F*_ST_ values between populations were low, but significant (*p* < 0.001) for all pairwise comparisons (electronic supplementary material, table S1). We found the lowest *F*_ST_ values among the rural populations (mean *F*_ST_ = 0.003), intermediate values for the urban-rural pairs (mean *F*_ST_ = 0.014) and the highest values between the urban populations (mean *F*_ST_ = 0.025; *t*-tests among these three groups: all *p* < 0.02). The genetic variance between urban and rural habitats is small, but significant (dbRDA: 1.2%; AMOVA: 0.9%). The genetic variance between rural populations is similar or somewhat higher (dbRDA: 3.7%; AMOVA: 0.7%), but the highest variance occurs between urban populations (dbRDA: 5.3%; AMOVA: 7%; electronic supplementary material, table S2).

The genetic population structure can be visualized by plotting all individuals on the first two PCs of the PCA (figure 2). Although the total variance explained by the first two PCs was low, figure 2 clearly distinguishes between the sampled populations and habitat affiliations. Whereas all three rural populations showed little differentiation and were clustered in the centre of the plot, the urban populations spread out in different directions. Individuals belonging to different cities are clearly separated, whereas individuals within cities cluster together on a single line. This is also true for the two urban sampling sites from Bahia Blanca BB1_urban_ and BB2_urban_, although they show different clustering on the third PC (electronic supplementary material, figure S5). A few individuals from the rural populations lie further from the centre on the line of their respective urban population (figure 2). This indicates that there is some dispersal/gene flow back from the urban populations to the adjacent rural ones. The different urban-rural population structure was also corroborated by the DAPC analysis, in which the first two discriminant axes accounted for more than 20% of the genetic variation (electronic supplementary material, figure S6).

**Figure 3.**
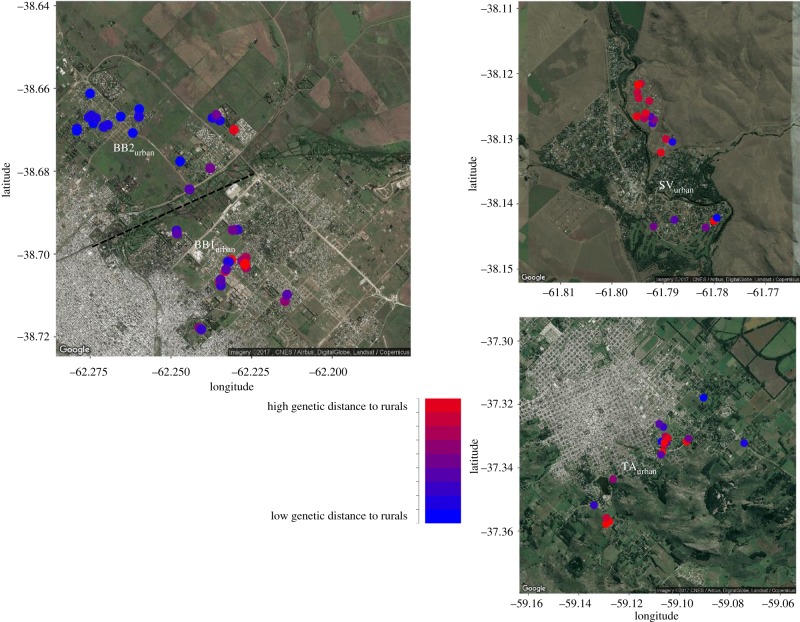
Maps showing the capture location of urban birds with dot colour reflecting the genetic distance to the rural populations. Colours range from blue (genetically closest to the rural centre, based on figure 2) to red (genetically most distant to the rural centre).

We further explored the spatial structure within urban and rural populations by testing isolation-by-distance for each population. As expected with some spatial structure, the correlation coefficients between identity-by-state values and geographical distance were negative in most populations (except SV_rural_; table 1). All negative coefficients were in the same range, except for the weaker correlation in SV_urban_. This indicates the same level of isolation-by-distance structure for all populations (except the SV populations), despite the strong difference in geographical scale (mean geographical distances among individuals) between urban and rural populations (table 1, electronic supplementary material, figure S7). The results of the dbRDA analysis (electronic supplementary material, table S2) are comparable to those of the Mantel tests (table 1): they indicate significant and similarly strong isolation-by-distance patterns within the urban populations (3.3% explained by longitude and latitude) and within the rural ones (3.7% explained; electronic supplementary material, table S2). This confirms that urban birds show substantial genetic variation over a small geographical scale (figure 3). Individuals with similar genetic distance to the rural birds are spatially clustered (figure 3), but individuals sampled closer to the city boundary do not show lower genetic distance than those sampled closer to the city centre (based on visual inspection of figure 3).

### (c) Demographic history

**Figure 4.**
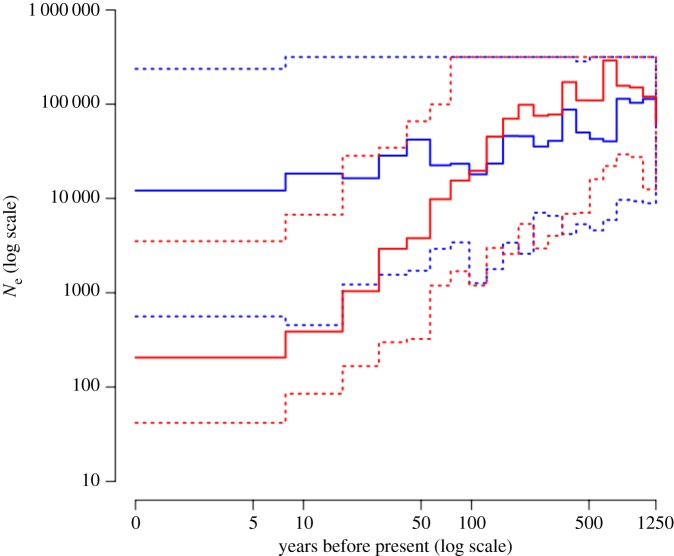
Estimated effective population size (*N*_e_) over the last 1250 years (500 generations) for all urban (red) and all rural (blue) populations combined. Median (solid line) and 90% credible intervals (dotted lines) of the 40 and 30 accepted simulations of the urban and rural populations, respectively.

Trajectories of estimated population sizes (*N*_e_) over the last 1250 years (500 generations) show clear and consistent differences between urban and rural populations, although the credibility intervals still overlap given the limited number of simulations (electronic supplementary material, figure S8). Whereas urban population size estimates decreased from approximately 100 000 to approximately 100 over the last 100 years, the rural population size estimates decreased only little or not at all over the same period. We thus combined the accepted ABC simulations of all urban populations and all rural populations and plotted median and credible intervals for the pooled groups (figure 4). Urban and rural populations show a shared demographic history until about 50–75 years ago.

## 4. Discussion

Using genome-wide SNP data from 137 burrowing owls from three urban-rural population pairs, we found a weak but significant population structure, whereby the three urban populations are genetically distinct from each other and from the rural populations (figure 2). The three widely distributed rural populations showed little differentiation (figure 2). The overall urban-rural population structure indicates that a limited number of founders from the surrounding rural area independently colonized the different cities and that there is no dispersal of individuals between cities. A similar colonization model has been described for the urbanization of the European blackbird [38]. The founder effect resulted in the random elimination of some rare SNPs, but it was too weak to affect the average heterozygosity of individuals in the urban populations. This confirms model predictions that founder events with subsequent restricted gene flow more often lead to loss of rare alleles than to a decrease in heterozygosity [39].

Among the urban birds, we found variation in their genetic distance from the cluster of rural birds (figure 2). On the other hand, some individuals from the rural populations did not belong to the rural cluster, i.e. they were more similar to individuals from the nearest urban population (figure 2). This indicates recent, but restricted gene flow between neighbouring urban and rural populations, whereby some urban birds recently emigrated to the rural population. Although ringing data of the Bahia Blanca population corroborate that there is limited gene flow between urban and rural sites, they suggest that rural birds also continue to move into the urban habitat: among 270 natal dispersal events, nine individuals (3%) ended up breeding in a habitat different than their natal one, whereby two moved from urban to rural, while seven moved from rural to urban habitat (our unpublished data).

Both rural and urban owl populations show signs of genetic isolation-by-distance at the individual level, indicating spatial restriction to gene flow even within the predefined populations. Note, however, that the spatial scale over which this isolation occurs differs: the strength of isolation-by-distance (as indicated by the correlation coefficients of the Mantel tests, table 1, and the proportion of genetic variance explained by the geographical positions of individuals within populations of the dbRDA analyses, electronic supplementary material, table S2) is comparable between the rural and urban birds, but the latter were sampled in a much more restricted area (figure 1 and table 1). This can be explained by more restricted gene flow in urban populations, where suitable habitat is more patchy and where barriers for moving between patches (e.g. streets, buildings) may exist even at small geographical scales. Such a pattern has been reported for several other species with lower mobility than birds, such as rodents [2,8]. Although unexpected given their high dispersal ability, weak but significant small-scale population structure related to urbanization has also been shown for other bird species, e.g. the house sparrow (*Passer domesticus*; [40,41]), the great tit (*Parus major*; [42]) and the Javan myna (*Acridotheres javanicus*; [43]). In our study model, reduced gene flow in the urban populations is more likely a consequence of a change in dispersal behaviour of urban individuals. Urban birds tend to be more philopatric and disperse shorter distances than rural individuals, and these differences in natal and breeding dispersal behaviour may be related to other differences in individual behaviour and to predation pressure (A. Luna *et al*. 2018, unpublished data). A similar difference in behaviour following urbanization, namely increased sedentariness and reduced migratory behaviour, has also been reported for the European blackbird [44,45]. Note that the city with the lowest level of isolation-by-distance (Sierra de la Ventana) is also the smallest and youngest settlement. Thus, burrowing owls may have colonized this city more recently and in lower numbers, so that there was less time and more limited genetic variability for genetic differentiation among the inhabitable city patches to develop. Our demographic inferences of the Sierra de la Ventana urban population do not support an earlier timing of colonization, but indicate a lower final effective population size in comparison to the other urban populations (electronic supplementary material, figure S8). The number of SNPs was also lower in this urban population (table 1). Alternatively, the lower level of urbanization in this city may have created fewer barriers to dispersal. A relationship between genetic differentiation and urban development age and intensity has also been described for the wrentit (*Chamaea fasciata*; [46]) and the song sparrow (*Melospiza melodia*; [47]).

At the between-population level, our results suggest that the genetically different urban populations of burrowing owls evolved independently under random genetic drift processes following limited founder populations and limited subsequent immigration. If so, the effective population size of urban populations should decrease from the moment of colonization onwards. We showed that the ABC approach proposed by Boitard *et al*. [13] using LD values including long-range values up to 17 Mb distance can consistently infer effective population sizes only 2.5 generations ago (= 6.25 years ago, assuming a generation time of 2.5 years). Most methods, e.g. the ones based on the Sequentially Markovian Coalescent model [48], are limited in the resolution of recent population size [13,49]. The estimated population size dynamics of urban and rural populations differ consistently. Whereas rural population size estimates remain more or less constant at values greater than 10 000 or show a moderate decline starting around 100 years ago, the estimated urban population sizes decrease consistently over the last 50–100 years to a current effective size between less than hundred to less than a thousand (electronic supplementary material, figure S8). The estimated start of the differentiation between urban and rural demographies lies around 50–75 years ago, which is consistent with the first observations of burrowing owls in Bahia Blanca and its suburbs more than 35 years ago (M. Carrete 2018, own observations).

Martinez *et al*. [14] hypothesized that burrowing owls started colonizing cities after they acquired the ability to dig their own burrows, which might have coincided with programmes to eradicate plains viscachas on agricultural land. Historically, burrowing owls typically used burrows of plains viscachas for breeding (hence the common Argentinian name of burrowing owls ‘lechucita de las vizcacheras'), which were still abundant at the end of the nineteenth century [50,51]. However, viscachas were declared a national pest in Argentina in 1907 and subjected to government control programmes. This species still is intensively persecuted through shooting, trapping, poisoning and fuming, and appreciated as bush meat in our study area. Although detailed data on the current distribution of the species is lacking, viscachas have long disappeared from most of the pampas [51]. The reduction in nest site availability at rural sites might have promoted the spread of the innovative digging behaviour of the burrowing owl which then served as pre-adaptation for the urban colonization.

Our data are consistent with the general pattern that urban populations are smaller and more genetically unique (owing to isolation with random genetic drift) than rural ones [2]. Genetic drift and isolation were not strong enough to decrease overall genetic diversity measured as heterozygosity, which is mostly influenced by common SNPs. However, the number of rare SNPs (singletons in the total dataset excluded) decreased by 4 to 12% in the urban populations in comparison to the neighbouring rural populations. Thus, initial selection after urbanization potentially acting on rare standing variation might be limited and variable between cities. This leads to the following considerations when designing genomic studies searching for signals of selection. Selection studies addressing common variants will benefit from having multiple replicates (here multiple urban-rural pairs), which allows scanning for consistent selection signals across these replicates. By contrast, selection signals based on rare variants should mostly rely on information from single populations (e.g. sampling over different time periods during the urbanization process). Noticeably, the few molecular studies investigating selection in urban populations have reported allele frequency changes at both rare and common alleles [52,53,54], but more work is needed to understand how selection acts on standing variation [55].

## Supplementary Material

Supplementary figures 1-8

## Supplementary Material

Supplementary tables 1-2

## Supplementary Material

Supplementary text S1

## Data accessibility

The burrowing owl genome browser can be found at http://public-genomes-ngs.molgen.mpg.de. The raw sequences and the variant call format (VCF) file with all filtered SNPs and genotypes of all individuals are deposited at the NCBI archive under bioproject no. PRJNA431202.

## References

[1] Sol D, González-Lagos C, Moreira D, Maspons J, Lapiedra O 2014 Urbanisation tolerance and the loss of avian diversity. Ecol. Lett. 17, 942–950. (10.1111/ele.12297) 24835452

[2] Johnson MTJ, Munshi-South J 2017 Evolution of life in urban environments. Science 358, eaam8327 (10.1126/science.aam8327) 29097520

[3] Carrete M, Tella JL 2011 Inter-individual variability in fear of humans and relative brain size of the species are related to contemporary urban invasion in birds. PLoS ONE 6, e18859 (10.1371/journal.pone.0018859) 21526193 PMC3079730

[4] Rebolo-Ifrán N, Carrete M, Sanz-Aguilar A, Rodríguez-Martínez S, Cabezas S, Marchant TA, Bortolotti GR, Tella JL 2015 Links between fear of humans, stress and survival support a non-random distribution of birds among urban and rural habitats. Sci. Rep. 5, 13723 (10.1038/srep13723) 26348294 PMC4562227

[5] Alberti M, Correa C, Marzluff JM, Hendry AP, Palkovacs EP, Gotanda KM, Hunt VM, Apgar TM, Zhou Y 2016 Global urban signatures of phenotypic change in animal and plant populations. Proc. Natl Acad. Sci. USA 114, 8951–8956. (10.1073/pnas.1606034114) PMC5576774 28049817

[6] Rebolo-Ifrán N, Tella JL, Carrete M 2017 Urban conservation hotspots: predation release allows the grassland-specialist burrowing owl to perform better in the city. Sci. Rep. 7, 3527 (10.1038/s41598-017-03853-z) 28615700 PMC5471179

[7] Marzluff JM 2016 A decadal review of urban ornithology and a prospectus for the future. Ibis 159, 1–13. (10.1111/ibi.12430)

[8] Munshi-South J, Zolnik CP, Harris SE 2016 Population genomics of the Anthropocene: urbanization is negatively associated with genome-wide variation in white-footed mouse populations. Evol. Appl. 9, 546–564. (10.1111/eva.12357) 27099621 PMC4831458

[9] Evans KL, Hatchwell BJ, Parnell M, Gaston KJ 2010 A conceptual framework for the colonization of urban areas: the blackbird *Turdus merula* as a case study. Biol. Rev. 85, 643–667. 20128785 10.1111/j.1469-185X.2010.00121.x

[10] Atwell JW, Cardoso GC, Whittaker DJ, Campbell-Nelson S, Robertson KW, Ketterson ED 2012 Boldness behavior and stress physiology in a novel urban environment suggest rapid correlated evolutionary adaptation. Behav. Ecol. 23, 960–969. (10.1093/beheco/ars059) 22936840 PMC3431113

[11] Edelaar P et al. 2015 Shared genetic diversity across the global invasive range of the monk parakeet suggests a common restricted geographic origin and the possibility of convergent selection. Mol. Ecol. 24, 2164–2176. (10.1111/mec.13157) 25873354

[12] Lawson DJ, Hellenthal G, Myers S, Falush D 2012 Inference of population structure using dense haplotype data. PLoS Genet. 8, e1002453 (10.1371/journal.pgen.1002453) 22291602 PMC3266881

[13] Boitard S, Rodríguez W, Jay F, Mona S, Austerlitz F 2016 Inferring population size history from large samples of genome-wide molecular data: an approximate Bayesian computation approach. PLoS Genet. 12, e1005877 (10.1371/journal.pgen.1005877) 26943927 PMC4778914

[14] Martinez G, Baladron AV, Cavalli M, Bo MS, Isacch JP 2017 Microscale nest-site selection by the burrowing owl (*Athene cunicularia*) in the pampas of Argentina. Wilson J. Ornithol. 129, 62–70. (10.1676/1559-4491-129.1.62)

[15] Rodríguez-Martínez S, Carrete M, Roques S, Rebolo-Ifrán N, Tella JL 2014 High urban breeding densities do not disrupt genetic monogamy in a bird species. PLoS ONE 9, e91314 (10.1371/journal.pone.0091314) 24614308 PMC3948869

[16] Carrete M, Martínez-Padilla J, Rodríguez-Martínez S, Rebolo-Ifrán N, Palma A, Tella JL 2016 Heritability of fear of humans in urban and rural populations of a bird species. Sci. Rep. 6, 31060 (10.1038/srep31060) 27499420 PMC4976307

[17] Carrete M, Tella JL 2017 Behavioral correlations associated with fear of humans differ between rural and urban burrowing owls. Front. Ecol. Evol. 5, 54 (10.3389/fevo.2017.00054)

[18] Peng Y, Leung HC, Yiu SM, Chin FY 2012 IDBA-UD: a de novo assembler for single-cell and metagenomic sequencing data with highly uneven depth. Bioinformatics 28, 1420–1428. (10.1093/bioinformatics/bts174) 22495754

[19] Iwata H, Gotoh O 2012 Benchmarking spliced alignment programs including Spaln2, an extended version of Spaln that incorporates additional species-specific features. Nucleic Acids Res. 40, e161 (10.1093/nar/gks708) 22848105 PMC3488211

[20] Langmead B, Salzberg S 2012 Fast gapped-read alignment with Bowtie 2. Nat. Methods 9, 357–359. (10.1038/nmeth.1923) 22388286 PMC3322381

[21] Van der Auwera GA et al. 2013 From FastQ data to high-confidence variant calls: the Genome Analysis Toolkit best practices pipeline. Curr. Protoc. Bioinformatics 43, 11.10.1–11.10.33. 25431634 10.1002/0471250953.bi1110s43 PMC4243306

[22] Danecek P et al. 2011 The variant call format and VCFtools. Bioinformatics 27, 2156–2158. (10.1093/bioinformatics/btr330) 21653522 PMC3137218

[23] Weir BS, Cockerham CC 1984 Estimating F-statistics for the analysis of population structure. Evolution 38, 1358–1370. 28563791 10.1111/j.1558-5646.1984.tb05657.x

[24] R Development Core Team. 2012 R: a language and environment for statistical computing. Vienna, Austria: R Foundation for Statistical Computing.

[25] Zheng X, Levine D, Shen J, Gogarten S, Laurie C, Weir B 2012 A high-performance computing toolset for relatedness and principal component analysis of SNP data. Bioinformatics 28, 3326–3328. (10.1093/bioinformatics/bts606) 23060615 PMC3519454

[26] Paradis E 2010 pegas: an R package for population genetics with an integrated–modular approach. Bioinformatics 26, 419–420. (10.1093/bioinformatics/btp696) 20080509

[27] Oksanen J et al 2018 vegan: community ecology package. R package version 2.4-6. See https://CRAN.R-project.org/package=vegan.

[28] Jombart T, Devillard S, Balloux F 2010 Discriminant analysis of principal components: a new method for the analysis of genetically structured populations. BMC Genet. 11, 94 (10.1186/1471-2156-11-94) 20950446 PMC2973851

[29] Karney CFF 2013 Algorithms for geodesics. J. Geod. 87, 43–55. (10.1007/s00190-012-0578-z)

[30] Goslee SC, Urban DL 2007 The ecodist package for dissimilarity-based analysis of ecological data. J. Stat. Softw. 22, 1–19. (10.18637/jss.v022.i07)

[31] Staab PR, Zhu S, Metzler D, Lunter G 2015 scrm: efficiently simulating long sequences using the approximated coalescent with recombination. Bioinformatics 31, 1680–1682. (10.1093/bioinformatics/btu861) 25596205 PMC4426833

[32] Nam K et al. 2010 Molecular evolution of genes in avian genomes. Genome Biol. 11, R68 (10.1186/gb-2010-11-6-r68) 20573239 PMC2911116

[33] Ellegren H 2013 The evolutionary genomics of birds. Annu. Rev. Ecol. Evol. Syst. 44, 239–259. (10.1146/annurev-ecolsys-110411-160327)

[34] Smeds L, Qvarnström A, Ellegren H 2016 Direct estimate of the rate of germline mutation in a bird. Genome Res. 26, 1211–1218. (10.1101/gr.204669.116) 27412854 PMC5052036

[35] Kardos M, Qvarnström A, Ellegren H 2017 Inferring individual inbreeding and demographic history from segments of identity by descent in *Ficedula* flycatcher genome sequences. Genetics 205, 1319–1334. (10.1534/genetics.116.198861) 28100590 PMC5340341

[36] Hayes BJ, Visscher PM, McPartlan HC, Goddard ME 2003 Novel multilocus measure of linkage disequilibrium to estimate past effective population size. Genome Res. 13, 635–643. (10.1101/gr.387103) 12654718 PMC430161

[37] Csillery K, Francois O, Blum MGB 2012 abc: an R package for approximate Bayesian computation (ABC). Methods Ecol. Evol. 3, 475–479. (10.1111/j.2041-210X.2011.00179.x)

[38] Evans KL et al. 2009 Independent colonization of multiple urban centres by a formerly forest specialist bird species. Proc. R. Soc. B 276, 2403–2410. (10.1098/rspb.2008.1712) PMC2691005 19364751

[39] Greenbaum G, Templeton AR, Zarmi Y, Bar-David S 2014 Allelic richness following population founding events: a stochastic modeling framework incorporating gene flow and genetic drift. PLoS ONE 9, e115203 (10.1371/journal.pone.0115203) 25526062 PMC4272294

[40] Vangestel C, Mergeay J, Dawson DA, Vandomme V, Lens L 2011 Spatial heterogeneity in genetic relatedness among house sparrows along an urban-rural gradient as revealed by individual-based analysis. Mol. Ecol. 20, 4643–4653. (10.1111/j.1365-294X.2011.05316.x) 22004175

[41] Vangestel C, Mergeay J, Dawson DA, Callens T, Vandomme V, Lens L 2012 Genetic diversity and population structure in contemporary house sparrow populations along an urbanization gradient. Heredity 109, 163–172. (10.1038/hdy.2012.26) 22588131 PMC3424918

[42] Perrier C, del Campo AL, Szulkin M, Demeyrier V, Gregoire A, Charmantier A 2017 Great tits and the city: distribution of genomic diversity and gene-environment associations along an urbanization gradient. Evol. Appl. 00, 1–21. (10.1111/eva.12580) PMC5979639 29875805

[43] Low GW, Chattopadhyay B, Garg KM, Irestedt M, Ericson PGP, Yap G, Tang Q, Wu S, Rheindt FE 2018 Urban landscape genomics identifies fine-scale gene flow patterns in an avian invasive. Heredity 120, 138–153. (10.1038/s41437-017-0026-1) 29225353 PMC5837122

[44] Partecke J, Gwinner E 2007 Increased sedentariness in European blackbirds following urbanization: a consequence of local adaptation? Ecology 88, 882–890. (10.1890/06-1105) 17536705

[45] Evans KL, Newton J, Gaston KJ, Sharp SP, McGowan A, Hatchwell BJ 2012 Colonisation of urban environments is associated with reduced migratory behaviour, facilitating divergence from ancestral populations. Oikos 121, 634–640. (10.1111/j.1600-0706.2011.19722.x)

[46] Delaney KS, Riley SPD, Fisher RN 2010 A rapid, strong, and convergent genetic response to urban habitat fragmentation in four divergent and widespread vertebrates. PLoS ONE 5, e12767 (10.1371/journal.pone.0012767) 20862274 PMC2940822

[47] Unfried TM, Hauser L, Marzluff JM 2013 Effects of urbanization on song sparrow (*Melospiza melodia*) population connectivity. Conserv. Genet. 14, 41–53. (10.1007/s10592-012-0422-2)

[48] Schiffels S, Durbin R 2014 Inferring human population size and separation history from multiple genome sequences. Nat. Genet. 46, 919–925. (10.1038/ng.3015) 24952747 PMC4116295

[49] Schraiber JG, Akey JM 2015 Methods and models for unravelling human evolutionary history. Nat. Rev. Genet. 16, 727–740. (10.1038/nrg4005) 26553329

[50] D'Orbigny A 1845 (Edición 2002). *Viaje a la América Meridional.* Tomo II. Plural Editores, La Paz, Bolivia.

[51] Branch L, Villareal D, Machicote M 2002 Conservation challenges of ecosystem engineers: case studies from grasslands and shrublands of North and South America. Open Ctry. 4, 37–48.

[52] Mueller JC, Partecke J, Hatchwell BJ, Gaston KJ, Evans KL 2013 Candidate gene polymorphisms for behavioural adaptations during urbanization in blackbirds. Mol. Ecol. 22, 3629–3637. (10.1111/mec.12288) 23495914

[53] van Dongen WFD, Robinson RW, Weston MA, Mulder RA, Guay P-J 2015 Variation at the DRD4 locus is associated with wariness and local site selection in urban black swans. BMC Evol. Biol. 15, 253 (10.1186/s12862-015-0533-8) 26653173 PMC4676183

[54] Harris SE, Munshi-South J 2017 Signatures of positive selection and local adaptation to urbanization in white-footed mice (*Peromyscus leucopus*). Mol. Ecol. 26, 6336–6350. (10.1111/mec.14369) 28980357 PMC5716853

[55] Barrett RDH, Schluter D 2007 Adaptation from standing genetic variation. Trends Ecol. Evol. 23, 38–44. (10.1016/j.tree.2007.09.008) 18006185

