# Supplementary material for: Evolution of genomic variation in the burrowing owl in response to recent colonization of urban areas
Source: Proc Biol Sci. 2018 May 16;285(1878):20180206. doi: 10.1098/rspb.2018.0206 (PMC5966595; doi:10.1098/rspb.2018.0206)
Supplement: Supplementary figures 1-8 [file rspb20180206supp1.pdf]

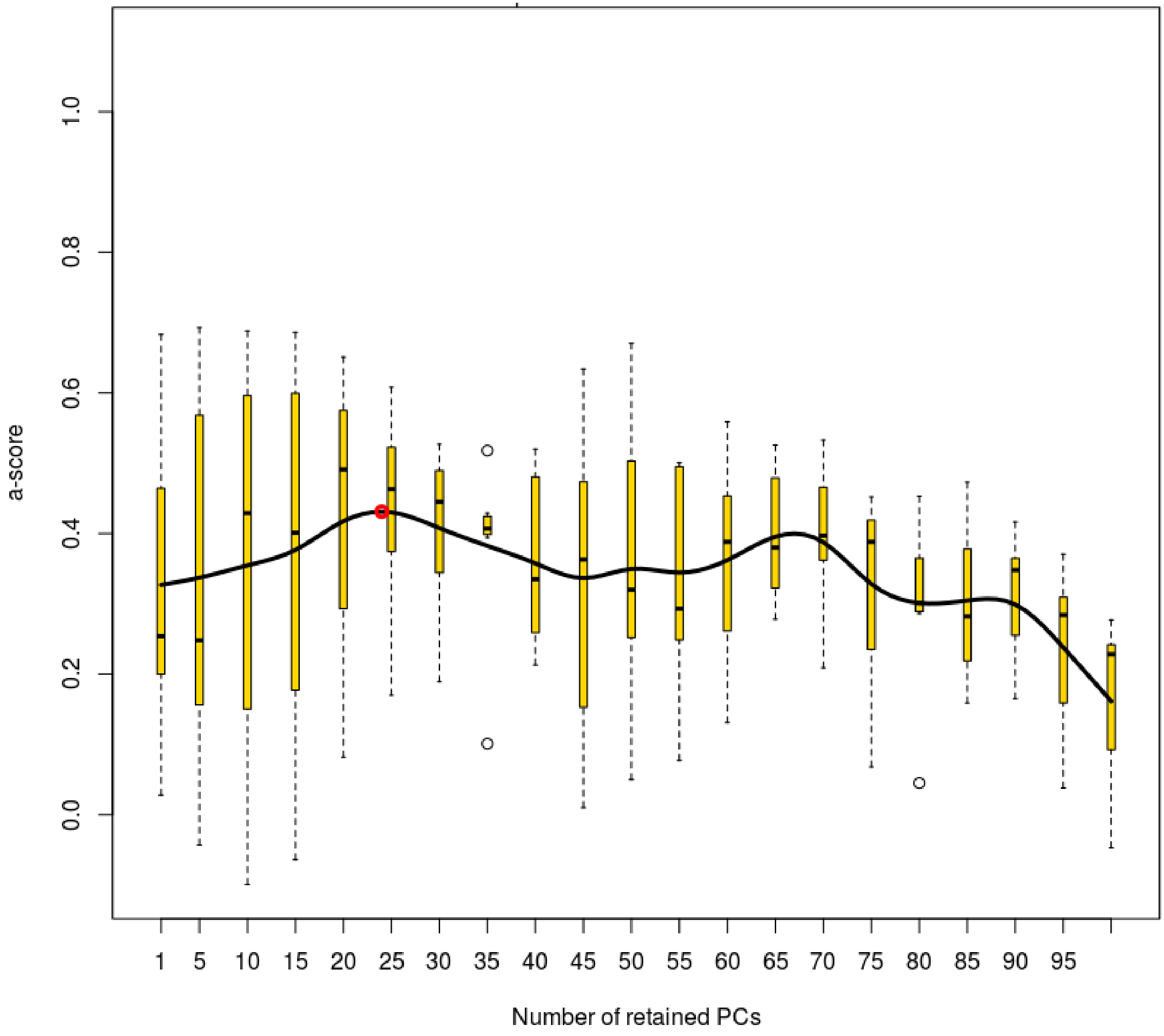

Supplementary figure 1. Plot of a-scores (probability of reclassification into the pre-specified populations minus probability of reclassification into randomly permuted clusters) along the number of retained PCs. Boxplots show the results of 50 DAPC simulations. Spline interpolation suggests 24 PCs as the optimal number for the DAPC analysis.

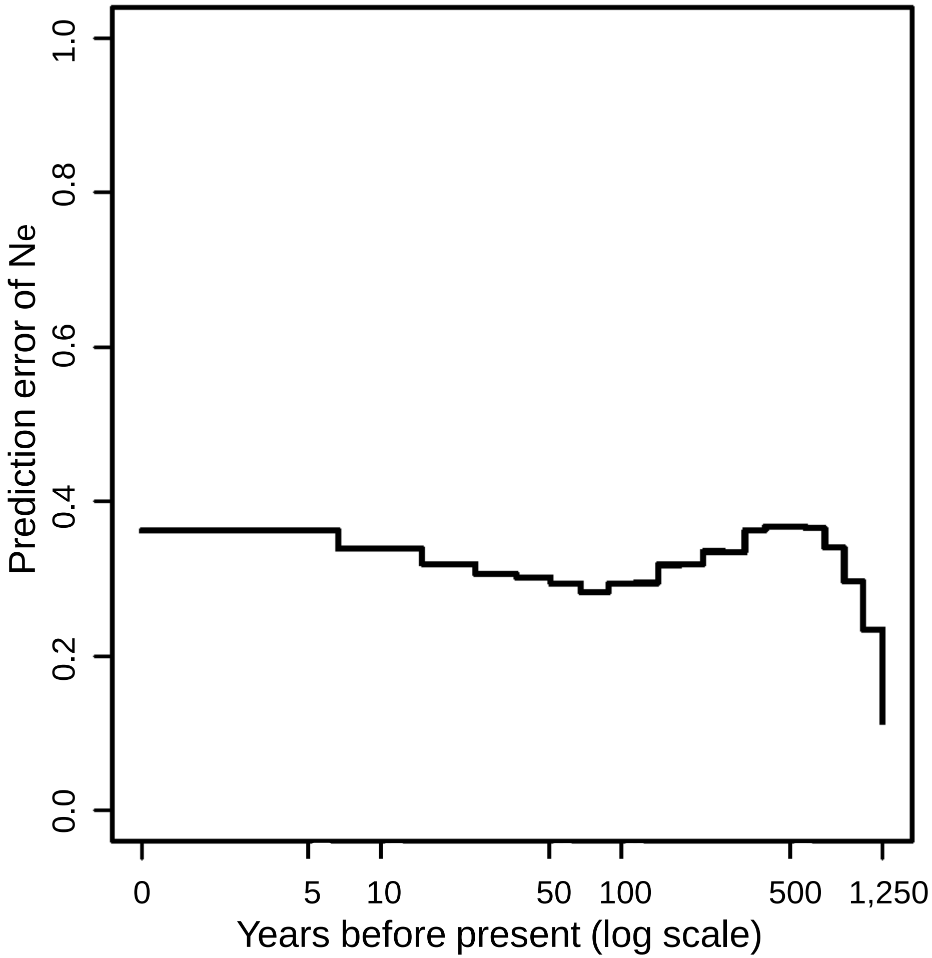

Supplementary figure 2. Prediction error for 2000 random samples of the 200,000 demographic simulations for each time interval

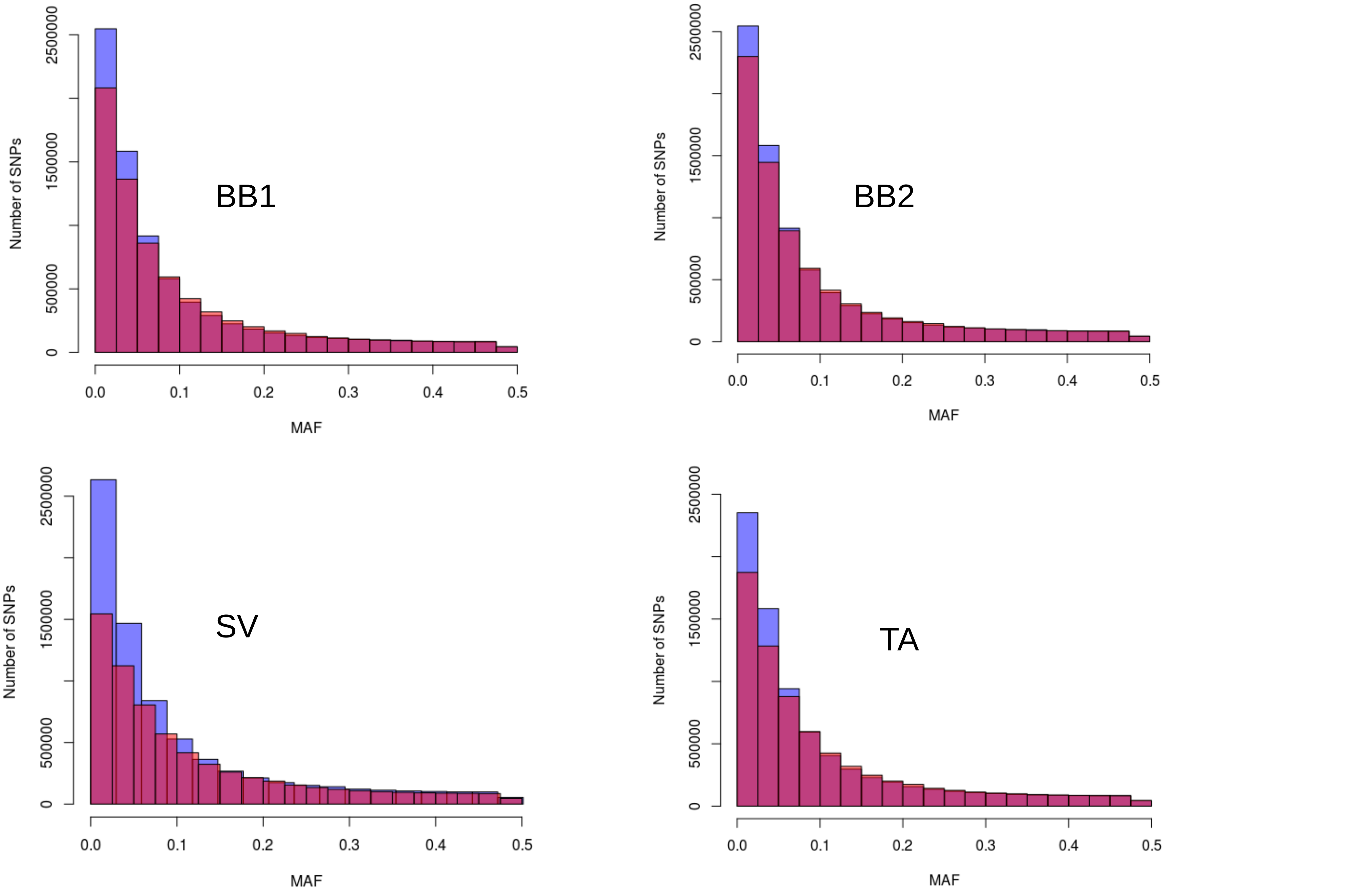

Supplementary figure 3. Comparison of minor allele frequency (MAF) distributions (number of SNPs) in urban-rural pairs. Each panel represents an overlay of the urban MAF histogram with the rural MAF histogram. Blue=SNP distribution surplus in the rural population; orange=SNP distribution surplus in the urban population; purple=SNP distribution overlap in both populations. The MAFs of the urban and rural population of SV are differently binned due to the different sample sizes.

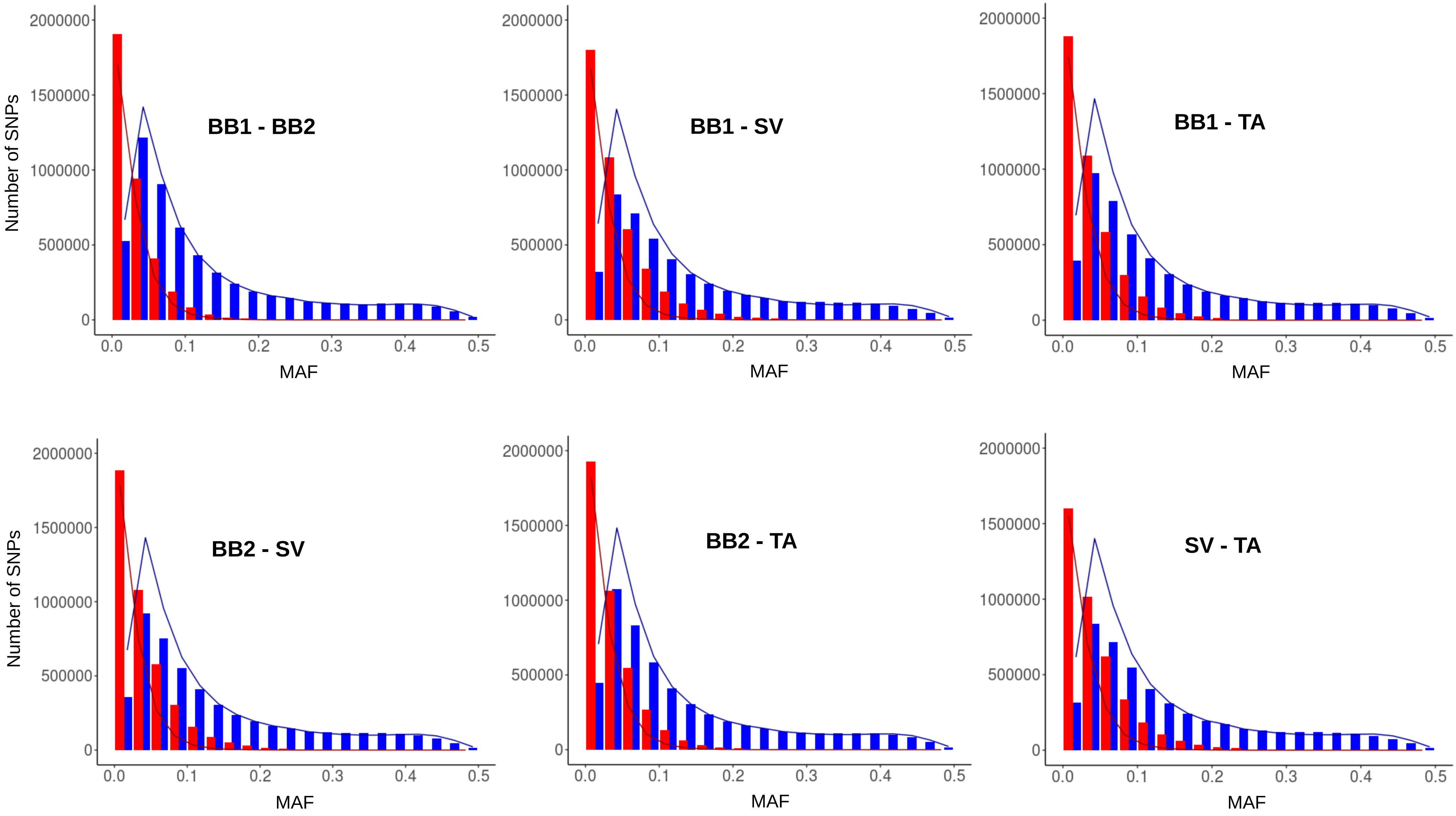

Supplementary figure 4: Comparison of the number of shared and non-shared SNPs along the minor allele frequency (MAF) classes in urban-urban pairs. Blue=shared SNPs; red=non-shared SNPs. The expected distributions of shared and non-shared SNP numbers under random sampling are indicated by the dark-blue and dark-red line, respectively.

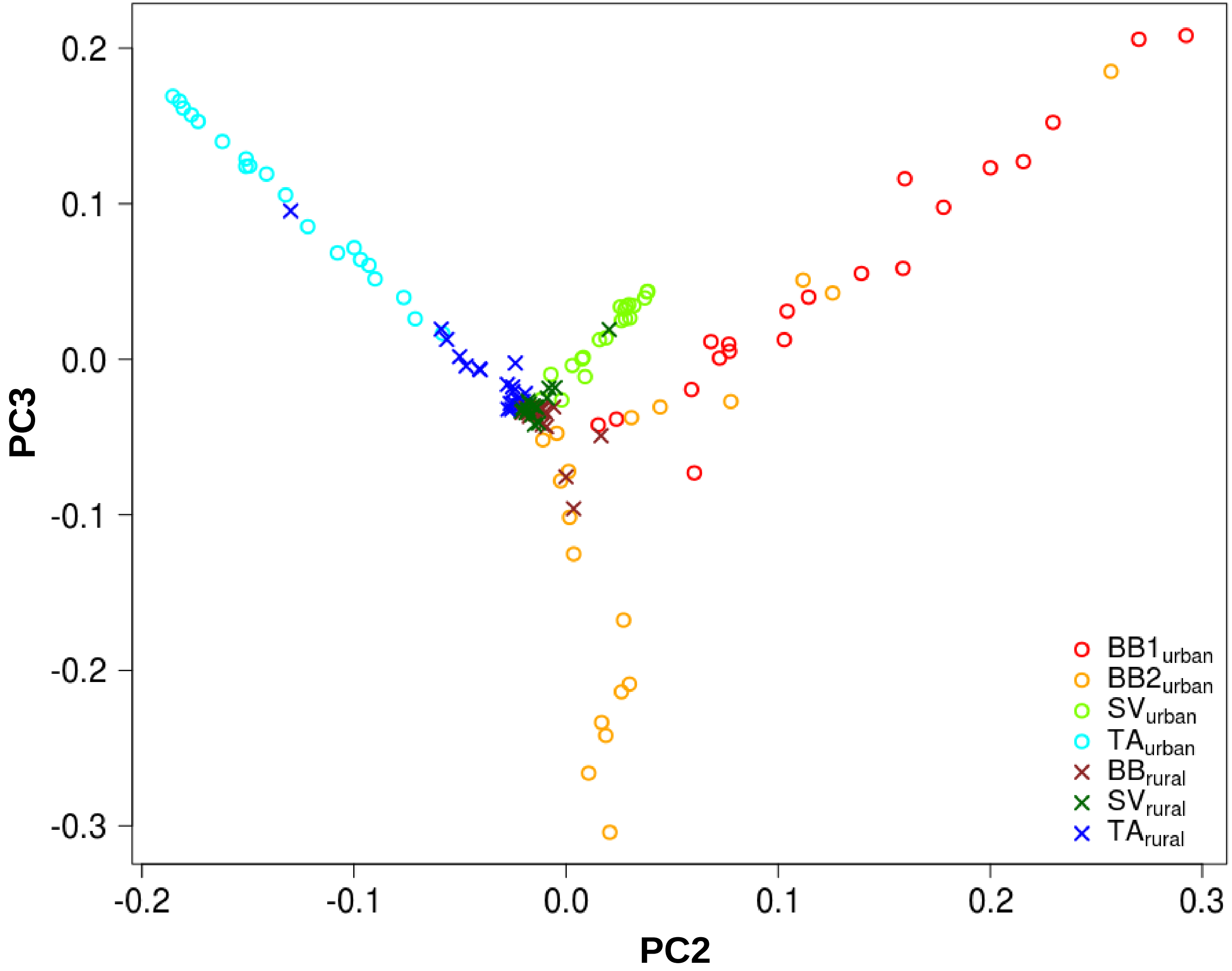

Supplementary figure 5. All individuals plotted on the second and third principal components of a PCA on SNP genotypes (see Methods). PC2 accounts for 1.4% and PC3 for 1.2% of the total variance.

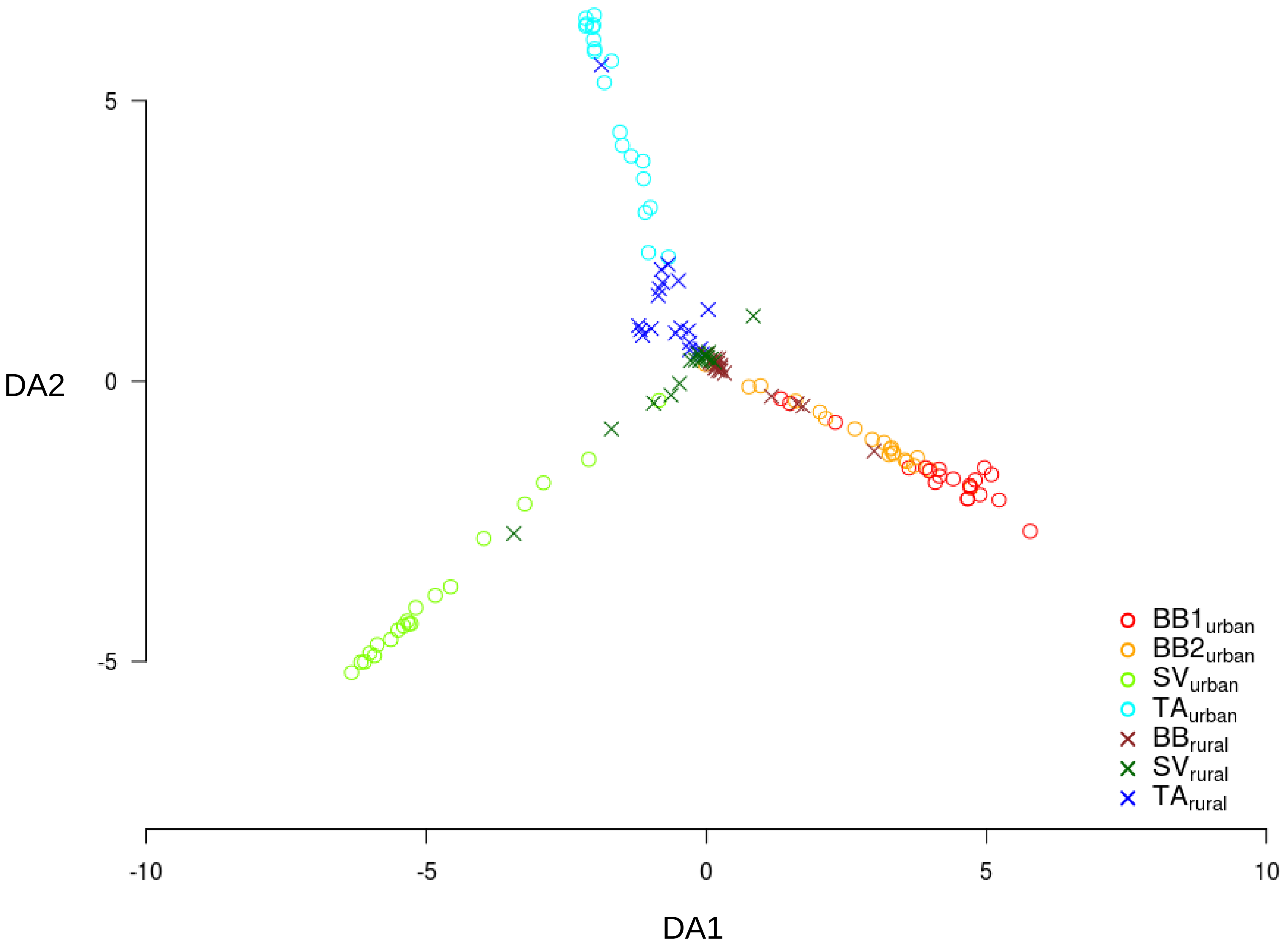

Supplementary figure 6. All individuals plotted on the first two discriminant axes of the DAPC on 24 PCs (26% cumulative variance). DA1 accounts for 43% and DA2 for 36% of captured variance.

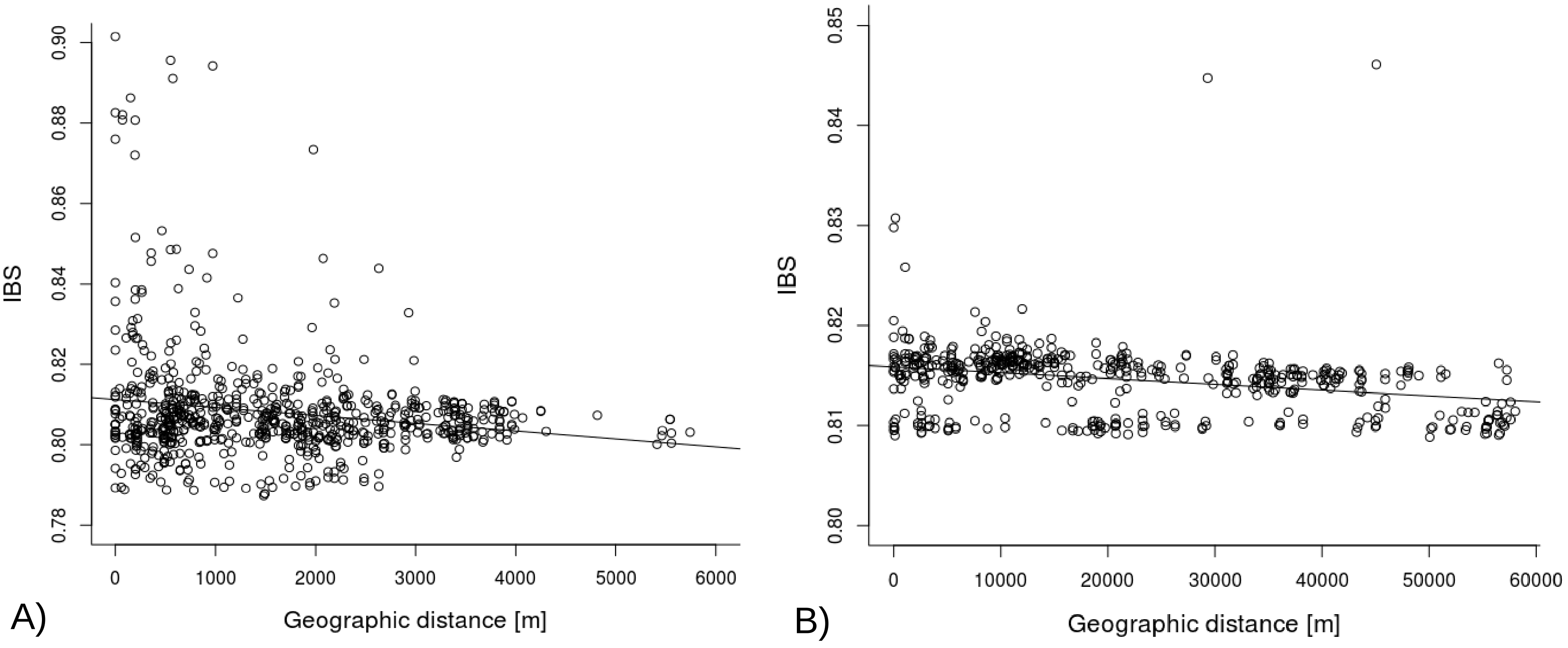

Supplementary figure 7. Correlation between identity-by-state (IBS) values and geographic distances within A) all urban populations and B) all rural populations. The regression line is added.

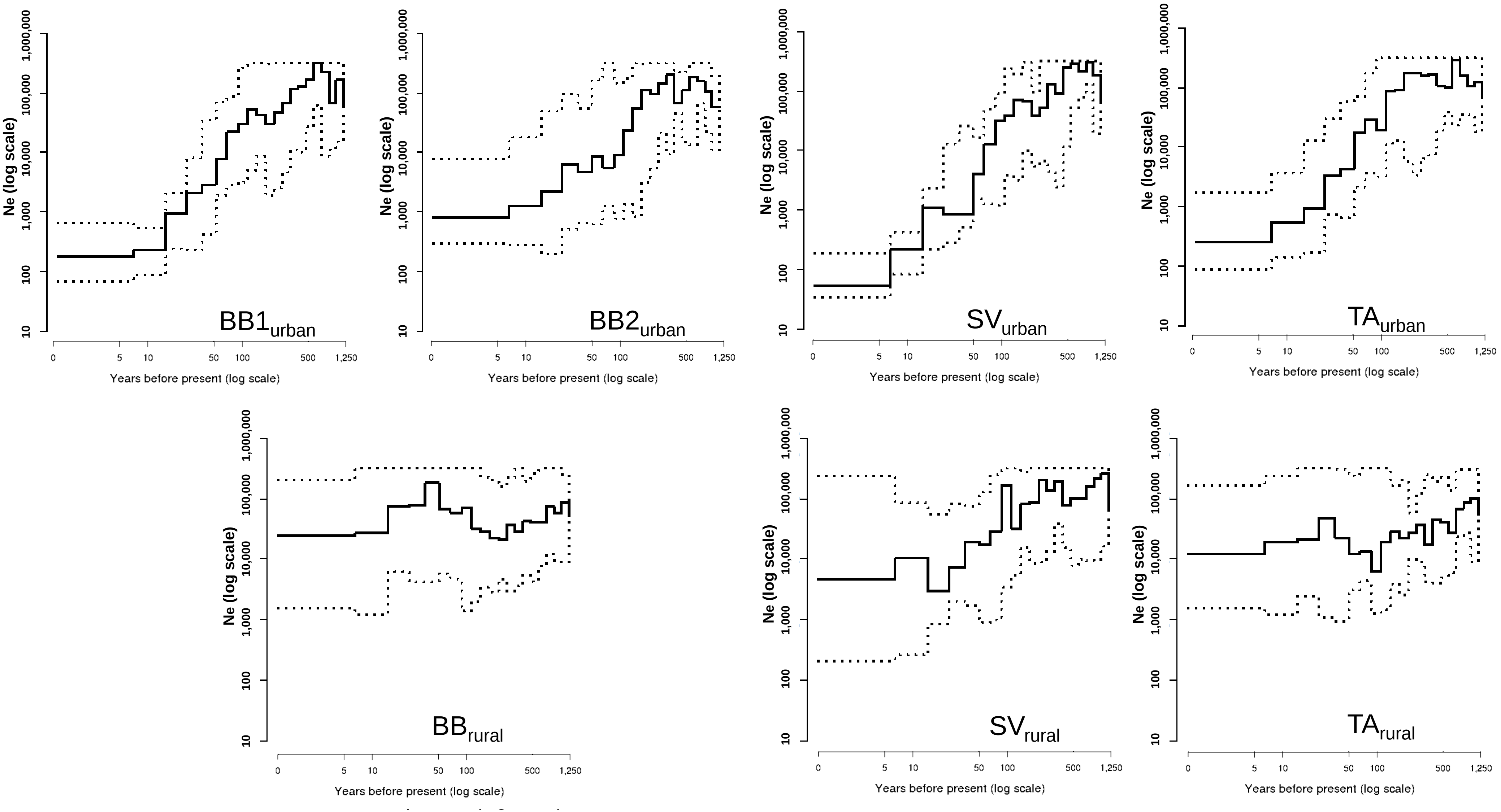

Supplementary figure 8. Estimated effective population size (Ne) over the last 1250 years (500 generations) of urban and rural populations. Median (solid line) and 90% credible interval (dotted lines) of the 10 accepted simulations.
