# Supplementary material for: Evolution of genomic variation in the burrowing owl in response to recent colonization of urban areas
Source: Proc Biol Sci. 2018 May 16;285(1878):20180206. doi: 10.1098/rspb.2018.0206 (PMC5966595; doi:10.1098/rspb.2018.0206)
Supplement: Supplementary tables 1-2 [file rspb20180206supp2.docx]

**Supplementary table 1.** F_ST_-values between all populations (sampling sites).

|  | BB1_urban_ | BB2_urban_ | SV_urban_ | TA_urban_ | BB_rural_ | SV_rural_ |
| --- | --- | --- | --- | --- | --- | --- |
| BB2_urban_ | 0.012 |  |  |  |  |  |
| SV_urban_ | 0.033 | 0.028 |  |  |  |  |
| TA_urban_ | 0.024 | 0.019 | 0.032 |  |  |  |
| BB_rural_ | 0.013 | 0.007 | 0.022 | 0.013 |  |  |
| SV_rural_ | 0.014 | 0.009 | 0.018 | 0.013 | 0.002 |  |
| TA_rural_ | 0.014 | 0.009 | 0.022 | 0.010 | 0.003 | 0.004 |

All F_ST_-values are significantly different from zero at p<0.001

**Supplementary table 2.** Proportion of variance and its significance based on RDA models and on AMOVA models. RDA models are conditioned on habitat, population or geographic positions of individuals, respectively. AMOVA models ignore geographic positions of individuals.

|  |  |  | RDA |  | AMOVA |  |
| --- | --- | --- | --- | --- | --- | --- |
|  | Constraint/effect | Condition | % variance | p | % variance | p |
| Urban and rural |  |  |  |  |  |  |
|  | Habitat | Longitude/latitude | 1.2 | <0.001 | 0.9 | <0.0001 |
|  | Population (within habitat) | Longitude/latitude/habitat | 5.1 | <0.001 | 4.5 | <0.0001 |
|  | Longitude/latitude | Habitat | 3.3 | <0.001 |  |  |
|  | Longitude/latitude | Population | 1.6 | 0.004 |  |  |
| Urban |  |  |  |  |  |  |
|  | Population | Longitude/latitude | 5.3 | <0.001 | 7.0 | <0.0001 |
|  | Longitude/latitude | Population | 3.3 | <0.001 |  |  |
| Rural |  |  |  |  |  |  |
|  | Population | Longitude/latitude | 3.7 | 0.026 | 0.7 | <0.0001 |
|  | Longitude/latitude | Population | 3.7 | 0.044 |  |  |
