# Supplementary material for: Evolution of genomic variation in the burrowing owl in response to recent colonization of urban areas
Source: Proc Biol Sci. 2018 May 16;285(1878):20180206. doi: 10.1098/rspb.2018.0206 (PMC5966595; doi:10.1098/rspb.2018.0206)
Supplement: Supplementary text S1 [file rspb20180206supp3.docx]

**Genome assembly and annotation** *(detailed information)*

We extracted DNA of the reference male blood sample using a standard phenol-chloroform protocol. The sample was used to construct a 500-bp insert Illumina Truseq2 paired-end sequencing library, a Nextera gel-free (no size selection) mate-pair library (insert size peak at about 2500-3500 bp) and a 10000-bp Nextera size-selected mate-pair library. DNA sequencing was performed using a 2 x 100 bp sequencing protocol on an Illumina Hiseq2500 system at the Sequencing Core Facility of the MPI for Molecular Genetics in Berlin.

Prior to assembly, adaptor removal and quality clipping as well as removal of PCR-duplicated read-pairs were performed on the paired-end reads using a custom script. In this regard, reads matching the first 16 bp of Illumina adaptors were removed. Quality clipping was performed to retain the longest sub-sequence of a read that contained base calls with quality scores better than Q11 and a minimum length of 32 bp. We only retained read pairs, single reads were discarded. Duplicate fragments were removed, if the first 32 bp of both reads in a read pair had been observed in another read-pair. Mate-pair reads were trimmed and de-duplicated using NextClip (version 1.1, Leggett et al. 2014). After the NextClip step, additional quality clipping and de-duplication was performed as described for the paired-end data.We obtained a total sequencing coverage of about 46-fold. Sequencing coverage was about 23-fold, 21-fold and 2-fold for the 500-bp insert paired-end, gel-free mate-pair and 10-kb mate-pair libraries, respectively. The high complexity of the gel-free mate-pair library provided a high physical (or fragment) coverage of the genome by mate pairs (about 250-fold).

We assembled the sequencing reads using the IDBA assembler (Peng et al. 2012) followed by the Newbler v3 assembler (Roche Diagnostics, Basel, Switzerland). This hybrid assembly strategy is taking advantage from combining de Bruijn graph (IDBA) and overlap consensus layout assembly strategies (Newbler). We compiled a tweaked version of IDBA which allowed for large kmer assembly (maximum kmer size 252 bp as described in <https://groups.google.com/forum/#!topic/hku-idba/p8YpZL46dtI> ). As IDBA performs local assemblies of paired-end reads and iterates from smaller to larger kmers, it is possible to use kmers larger than the read length for assembly. IDBA local-contigs resulting from local assembly (assembling reads that anchor with one read at the end of a contig and the other read outside the contig) are concatenated and separated by “n” characters, thus we splitted these sequences at character “n”. Contigs that were exceeding the maximum read length limit of Newbler3 were splitted into 29 kb fragments with 4 kb overlap. The processed IDBA (local)-contigs from kmer sizes 206, 226 and 246 bp were converted to fastq format with base quality set to Q40 and used for OLC assembly in Newbler3, which improved N50 contig length.

Long-range continuity (scaffold N50) was improved using the mate-pair information with SSPACE2.0 (Boetzer et al. 2011) and by comparison with the Gallus gallus version 4 genome. Three scaffolding runs using the SSPACE scaffolder were performed on Newbler v3 output (contigs larger than 2 kb) to iteratively make use of the long range information present in the non-size-selected mate-pair library. During the first run the median insert size of this library was set to the values observed in the distribution peaks and to a minimum required link count of 16. For the second run the scaffolds from the first run were re-scaffolded using a median insert size setting of 7000 bp and a minimum link count of 8. The third round used 14 kb as median insert size setting and a minimum of 4 required links. For the comparison with the *Gallus gallus* genome, we used LAST whole-genome alignment (Kiełbasa et al. 2011), filtered for 1:1 orthologous sequence alignments using single_cov2 (in the MULTIZ/TBA software package; Blanchette et al. 2004) and built chromosomal-sized superscaffolds with the software Ragout, version 1.0 (Kolmogorov et al. 2014, note that v1.0 allows directly using MAF alignment files, this was removed in later versions). The LAST/Ragout steps were performed a second time after remapping all mate-pair reads to the superscaffolds using Bowtie2 (Langmead and Salzberg 2012), calculating physical coverage of consistently mapped reads (reverse/forward orientation, span between 1 and 24 kb) by the bedtools “genomcov” option and by splitting the scaffolds at potential mis-assemblies (uniquely mapped mate pair physical coverage < 5). Finally, the assembly short-range continuity (contig N50) was improved using the GapCloser tool (Luo et al. 2012).

We annotated protein-coding sequences (cds) by alignment of all avian protein sequences of the NCBI protein database (date: 2015-09-22; 1,786,861 proteins) against the burrowing owl genome using SPALN v2.1.2 (Iwata and Gotoh 2012). SPALN GFF3 output was converted to GTF format by custom scripts. To choose the best SPALN gene models, we first filtered for gene predictions whose exons were supported by at least 3 other SPALN predictions. Subsequently, we chose the best scoring model for each gene locus by removing all cds models that had at least one exact exon match on the same strand to a higher scoring cds model and in a second iteration by removing residual models with non-exact exon overlaps on the same strand. Procedures to select the best gene models often results in loss of some gene models. We manually checked the results (17,858 cds models with id “BP_...”) and re-incorporated 304 models (id “P_...”) that had been removed due to only slight redundancies. The functional annotation of the resulting 18,162 cds models corresponds to the gene description, source organism and accession of the aligned NCBI protein from which the model was deduced.

The assembly and annotation are available through a custom installation of the UCSC genome browser (Kent et al. 2002).

**References**

Blanchette M, Kent WJ, Riemer C, Elnitski L, Smit AFA, Roskin KM, Baertsch R, Rosenbloom K, Clawson H, Green ED, Haussler D, and Miller W 2004. Aligning multiple genomic sequences with the threaded blockset aligner. Genome Research 14, 708–715.

Boetzer M, Henkel CV, Jansen HJ, Butler D, Pirovano W 2011. Scaffolding pre-assembled contigs using SSPACE. Bioinformatics 27, 578-579.

Iwata H, Gotoh O 2012. Benchmarking spliced alignment programs including Spaln2, an extended version of Spaln that incorporates additional species-specific features. Nucleic Acids Res. 40, e161.

Kent WJ, Sugnet CW, Furey TS, Roskin KM, Pringle TH, Zahler AM, Haussler D 2002. The human genome browser at UCSC. Genome Res. 12, 996-1006.

Kiełbasa SM, Wan R, Sato K, Horton P, Frith MC 2011. Adaptive seeds tame genomic sequence comparison. Genome Res. 21, 487-93.

Kolmogorov M, Raney B, Paten B, Pham S 2014. Ragout: a reference-assisted assembly tool for bacterial genomes. Bioinformatics 30, i302-309.

Langmead B, Salzberg S 2012. Fast gapped-read alignment with Bowtie 2. Nature Methods 9, 357-359.

Leggett RM, Clavijo BJ, Clissold L, Clark MD, Caccamo M 2014. NextClip: an analysis and read preparation tool for Nextera long mate pair libraries. Bioinformatics 30, 566-568.

Luo R, Liu B, Xie Y, Li Z, Huang W, Yuan J, He G, Chen Y, Pan Q, Liu Y, et al. 2012. SOAPdenovo2: an empirically improved memory-efficient short-read de novo assembler. GigaScience 1, 18.

Peng Y, Leung HC, Yiu SM, Chin FY 2012. IDBA-UD: a de novo assembler for single-cell and metagenomic sequencing data with highly uneven depth. Bioinformatics 28, 1420-1428.
